# Perioperative Complications and In-Hospital Mortality in Partial and Radical Nephrectomy Patients with Heart-Valve Replacement

**DOI:** 10.1245/s10434-024-15228-6

**Published:** 2024-03-25

**Authors:** Carolin Siech, Andrea Baudo, Mario de Angelis, Letizia Maria Ippolita Jannello, Francesco Di Bello, Jordan A. Goyal, Zhe Tian, Fred Saad, Shahrokh F. Shariat, Nicola Longo, Luca Carmignani, Ottavio de Cobelli, Alberto Briganti, Marina Kosiba, Philipp Mandel, Luis A. Kluth, Felix K. H. Chun, Pierre I. Karakiewicz

**Affiliations:** 1 Cancer Prognostics and Health Outcomes Unit, Division of Urology, University of Montréal Health Center, Montreal, Québec Canada; 2 Goethe University Frankfurt, University Hospital, Department of Urology, Frankfurt am Main, Germany; 3 Department of Urology, IRCCS Policlinico San Donato, Milan, Italy; 4 Vita-Salute San Raffaele University, Milan, Italy; 5 Division of Experimental Oncology/Unit of Urology, URI, IRCCS Ospedale San Raffaele, Milan, Italy; 6 Department of Urology, IEO European Institute of Oncology, IRCCS, Milan, Italy; 7 Università degli Studi di Milano, Milan, Italy; 8 Department of Neurosciences, Science of Reproduction and Odontostomatology, University of Naples Federico II, Naples, Italy; 9 Department of Urology, Comprehensive Cancer Center, Medical University of Vienna, Vienna, Austria; 10 Department of Urology, Weill Cornell Medical College, New York, NY USA; 11 Department of Urology, University of Texas Southwestern Medical Center, Dallas, TX USA; 12 Hourani Center for Applied Scientific Research, Al-Ahliyya Amman University, Amman, Jordan; 13 Department of Urology, IRCCS Ospedale Galeazzi – Sant’Ambrogio, Milan, Italy; 14 Department of Oncology and Haemato-Oncology, Università degli Studi di Milano, Milan, Italy

**Keywords:** Comorbidities, Heart surgery, Heart-valve replacement, Kidney cancer, NIS

## Abstract

**Background:**

In-hospital mortality and complication rates after partial and radical nephrectomy in patients with history of heart-valve replacement are unknown.

**Patients and Methods:**

Relying on the National Inpatient Sample (2000–2019), kidney cancer patients undergoing partial or radical nephrectomy were stratified according to presence or absence of heart-valve replacement. Multivariable logistic and Poisson regression models addressed adverse hospital outcomes.

**Results:**

Overall, 39,673 patients underwent partial nephrectomy versus 94,890 radical nephrectomy. Of those, 248 (0.6%) and 676 (0.7%) had a history of heart-valve replacement. Heart-valve replacement patients were older (median partial nephrectomy 69 versus 60 years; radical nephrectomy 71 versus 63 years), and more frequently exhibited Charlson comorbidity index ≥ 3 (partial nephrectomy 22 versus 12%; radical nephrectomy 32 versus 23%). In partial nephrectomy patients, history of heart-valve replacement increased the risk of cardiac complications [odds ratio (OR) 4.33; *p* < 0.001), blood transfusions (OR 2.00; *p* < 0.001), intraoperative complications (OR 1.53; *p* = 0.03), and longer hospital stay [rate ratio (RR) 1.25; *p* < 0.001], but not in-hospital mortality (*p* = 0.5). In radical nephrectomy patients, history of heart-valve replacement increased risk of postoperative bleeding (OR 4.13; *p* < 0.001), cardiac complications (OR 2.72; *p* < 0.001), intraoperative complications (OR 1.53; *p* < 0.001), blood transfusions (OR 1.27; *p* = 0.02), and longer hospital stay (RR 1.12; *p* < 0.001), but not in-hospital mortality (*p* = 0.5).

**Conclusions:**

History of heart-valve replacement independently predicted four of twelve adverse outcomes in partial nephrectomy and five of twelve adverse outcomes in radical nephrectomy patients including intraoperative and cardiac complications, blood transfusions, and longer hospital stay. Conversely, no statistically significant differences were observed in in-hospital mortality.

**Supplementary Information:**

The online version contains supplementary material available at 10.1245/s10434-024-15228-6.

Partial nephrectomy and radical nephrectomy represent guideline-recommended standard treatments in kidney cancer patients.^1,2^ Some patients who might benefit from partial or radical nephrectomy have a history of heart-valve replacement, which may predispose them to adverse in-hospital outcomes and possibly even higher in-hospital mortality. However, actual in-hospital complication rates and mortality figures in patients with history of heart-valve replacement treated with either partial or radical nephrectomy are unknown.

We addressed this knowledge gap and hypothesized that in-hospital outcomes, namely length of stay, estimated hospital cost, intraoperative and postoperative complications, critical care therapy use, and in-hospital mortality of partial or radical nephrectomy patients do not differ according to presence versus absence of history of heart-valve replacement in patients with kidney cancer. To test this hypothesis, we relied on a large-scale population-based cohort of kidney cancer patients who underwent partial or radical nephrectomy within the United States of America over a period of 20 years (2000–2019).

## Patients and Methods

### Data Source

Relying on discharge data from the National Inpatient Sample (NIS 2000–2019), we assessed length of stay, estimated hospital cost, perioperative complications, and in-hospital mortality of patients treated with partial or radical nephrectomy. NIS is a set of longitudinal hospital inpatient databases included in the Healthcare Cost and Utilization Project (HCUP) and formed by the Agency for Healthcare Research and Quality (AHRQ) through a Federal-State-Industry partnership.^3^ All diagnoses and procedures were coded using the International Classification of Disease (ICD) 9th revision Clinical Modification (ICD-9-CM), ICD 10th revision Clinical Modification (ICD-10-CM), as well as ICD 10th revision Procedure Coding System (ICD-10-PCS).

### Study Population

We included patients aged ≥ 18 years with a primary diagnosis of kidney cancer (ICD-9-CM code 189.0, and ICD-10-CM codes C64.1, C64.2, and C64.9). Only patients treated with partial (ICD-9 code 55.4, and ICD-10-PCS codes 0TB00ZZ, 0TB03ZZ, 0TB04ZZ, 0TB07ZZ, 0TB08ZZ, 0TB10ZZ, 0TB13ZZ, 0TB14ZZ, 0TB17ZZ, and 0TB18ZZ) or radical nephrectomy (ICD-9 codes 55.51 and 55.52, and ICD-10-PCS codes 0TT00ZZ, 0TT04ZZ, 0TT10ZZ, and 0TT14ZZ) were included.^4–7^ Bilateral nephrectomy represents a rare event and was therefore excluded from the current study. Patients were stratified according to history of heart-valve replacement (ICD-9-CM codes V42.2 and V43.3, and ICD-10-CM codes Z95.2–Z95.4).^8^

### Definition of Variables for Analyses

Study endpoints included length of stay, intraoperative and postoperative complications (bleeding, cardiac complications, pulmonary complications, vascular complications, gastrointestinal complications, and infections), blood transfusions,^4–7^ in-hospital mortality, and use of critical care therapies, defined as invasive mechanical ventilation, percutaneous endoscopic gastrostomy tube insertion, dialysis for acute kidney failure, total parenteral nutrition, and tracheostomy identified by ICD-9 and ICD-10 codes according to previously established methodology.^9–11^ All ICD-9 and ICD-10 codes used for the identification of intraoperative and postoperative complications are summarized in Supplementary Table 1. Additionally, estimated hospital cost were calculated relying on total hospital charges provided by NIS. Converting total hospital charges into estimated hospital cost was performed using HCUP Cost-to-Charge Ratios, which were based on hospital accounting reports, according to NIS methodological guidelines.^3,6^ All calculations were adjusted to 2019 US dollar ($USD) relying on the overall Consumer Price index.^12^ To account for comorbidities, the Deyo modification of Charlson comorbidity index (CCI) was used,^13^ according to coding algorithms for defining comorbidities in ICD-9-CM and ICD-10-CM codes by Quan et al.^14^. Covariates consisted of patient characteristics including age at admission (years, continuously coded) and CCI (0 versus 1 versus 2 versus ≥ 3).

### Statistical Analyses

First, descriptive characteristics and primary outcome rates were tabulated. For categorical variables, frequencies and proportions were reported. For continuously coded variables, medians and interquartile ranges (IQR) were reported. Wilcoxon rank sum test, Pearson’s chi-squared test, and Fisher’s exact test were applied. Second, estimated annual percentage changes (EAPC) were tested with the least squares linear regression. Third, univariable and multivariable Poisson regression models addressing length of stay and estimated hospital cost, as well as logistic regression models addressing perioperative complications and in-hospital mortality were fitted after adjustment for clustering at the hospital level using generalized estimating equation methodology.^10,11^ Subgroup analyses addressed patients with versus without history of prosthetic heart-valve replacement. All analytical steps were separately performed in partial nephrectomy and radical nephrectomy patients.

Analyses and reporting followed NIS reporting guidelines.^3^ Due to NIS data reporting agreement, counts and associated proportions were reported as less than eleven for sample sizes of less than eleven patients. R software environment was used for statistical computing and graphics (R version 4.2.2; R Foundation for Statistical Computing, Vienna, Austria).^15^ All tests were two sided, with a significance level set at *p* < 0.05.

## Results

### Descriptive Characteristics of the Study Population

Within NIS (2000–2019), we identified 39,673 kidney cancer patients who underwent partial nephrectomy, and 94,890 who underwent radical nephrectomy (Table 1). Of those, 248 (0.6%) and 676 (0.7%) had a history of heart-valve replacement. Over time, the annual proportions of patients with history of heart-valve replacement ranged from 0.3 to 1.4% in partial nephrectomy [EAPC −1.8%, 95% confidence interval (CI) −5.4 to +1.7%; *p* = 0.3] and from 0.5 to 1.1% in radical nephrectomy patients (EAPC +2.6%, 95% CI +1.1 to +4.0%; *p* = 0.003). History of heart-valve replacement patients were older (median age partial nephrectomy 69 versus 60 years; radical nephrectomy 71 versus 63 years), more frequently male (partial nephrectomy 80 versus 62%; radical nephrectomy 70 versus 62%), and more frequently exhibited CCI ≥3 (partial nephrectomy 22 versus 12%; radical nephrectomy 32 versus 23%).

**Table 1:** Descriptive characteristics of 134,563 kidney cancer patients, undergoing partial or radical nephrectomy, stratified according to history of heart-valve replacement

Characteristic		Partial nephrectomy, *n* = 39,673			Radical nephrectomy, *n* = 94,890		
		Heart-valve replacement, *n* = 248 (0.6%)	No heart-valve replacement, *n* = 39,425 (99.4%)	*p* value^a^	Heart-valve replacement, *n* = 676 (0.7%)	No heart-valve replacement, *n* = 94,214 (99.3%)	*p* value^a^
Age at admission, median (interquartile range in years)		69 (60, 76)	60 (51, 68)	**< 0.001**	71 (64, 78)	63 (54, 72)	**< 0.001**
Male sex, *n* (%)		199 (80%)	24,400 (62%)	**< 0.001**	476 (70%)	58,503 (62%)	**< 0.001**
Charlson comorbidity index, *n* (%)	0	83 (33%)	20,605 (52%)	**< 0.001**	192 (28%)	41,618 (44%)	**< 0.001**
	1	74 (30%)	9335 (24%)		156 (23%)	19,538 (21%)	
	2	37 (15%)	4844 (12%)		113 (17%)	10,993 (12%)	
	≥ 3	54 (22%)	4641 (12%)		215 (32%)	22,065 (23%)	
Hospital region, *n* (%)	West	50 (20%)	6999 (18%)	**0.004**	120 (18%)	18,247 (19%)	**0.04**
	Midwest	69 (28%)	8553 (22%)		176 (26%)	21,414 (23%)	
	Northeast	65 (26%)	9454 (24%)		136 (20%)	16,965 (18%)	
	South	64 (26%)	14,419 (37%)		244 (36%)	37,588 (40%)	
Teaching hospital status, *n* (%)		194 (78%)	30,442 (77%)	0.7	441 (65%)	59,249 (63%)	0.2
Hospital size, *n* (%)	Large (≥ 400 beds)	169 (68%)	26,788 (68%)	0.9	456 (68%)	62,403 (66%)	0.9
	Medium (200–399 beds)	49 (20%)	8204 (21%)		150 (22%)	21,572 (23%)	
	Small (< 200 beds)	30 (12%)	4312 (11%)		67 (10%)	9963 (11%)	

Bold values indicate statistically significant *p* < 0.05

^a^Wilcoxon rank sum test; Pearson’s chi-squared test

### Length of Stay, Estimated Hospital Cost, Perioperative Complications, and Mortality Rates in Partial Nephrectomy Patients

In partial nephrectomy patients (*n* = 39,673) with history of heart-valve replacement versus others, median length of stay was 4 versus 3 days (IQR 3–7 versus 2–5 days; *p* < 0.001; Table 2) and estimated median hospital cost were 46,133 versus 42,004 $USD (IQR 32,154–66,904 versus 29,671–62,873 $USD; *p* = 0.04). Addressing perioperative complications, 70 versus 2255 (28.2% versus 5.7%; *p* < 0.001) experienced cardiac complications, 29 versus 2663 (11.7% versus 6.8%; *p* = 0.002) intraoperative complications, 20 versus 3025 (8.1% versus 7.7%; *p* = 0.8) gastrointestinal complications, 17 versus 2863 (6.9 versus 7.3%; *p* = 0.8) pulmonary complications, < 11 versus 102 (< 4.4% versus 0.3%; *p* = 0.5) postoperative bleeding, < 11 versus 370 (< 4.4% versus 0.9%; *p* = 0.03) vascular complications, < 11 versus 239 (< 4.4% versus 0.6%; *p* = 0.2) infections, 40 versus 2676 (16.1 versus 6.8%; *p* < 0.001) received blood transfusions, and < 11 versus 628 (< 4.4% versus 1.6%; *p* = 0.1) received critical care therapy. In-hospital mortality was recorded in < 11 versus 97 (< 4.4 versus 0.2%; *p* = 0.1) patients. For five of the above outcomes, actual counts and actual proportions are not shown due to NIS reporting rules. Instead, counts of less than eleven and the associated proportions are shown.

**Table 2:** Perioperative length of stay, complications, and in-hospital mortality rates after partial or radical nephrectomy in 134,563 kidney cancer patients, stratified according to history of heart-valve replacement

Characteristic	Partial nephrectomy, *n* = 39,673			Radical nephrectomy, *n* = 94,890		
	Heart-valve replacement, *n* = 248 (0.6%)	No heart-valve replacement, *n* = 39,425 (99.4%)	*p* value^a^	Heart-valve replacement, *n* = 676 (0.7%)	No heart-valve replacement, *n* = 94,214 (99.3%)	*p* value^a^
Length of stay, median (interquartile range in days)	4 (3, 7)	3 (2, 5)	**< 0.001**	5 (3, 8)	4 (3, 6)	**< 0.001**
Estimated hospital cost, median (interquartile range in $USD)	46,133 (32,154, 66,904)	42,004 (29,671, 62,873)	**0.04**	47,439 (31,545, 72,111)	38,592 (26,036, 60,917)	**< 0.001**
Intraoperative complications, *n* (%)	29 (11.7%)	2663 (6.8%)	**0.002**	104 (15.3%)	8763 (9.3%)	**< 0.001**
*Postoperative complications*						
Bleeding, *n* (%)	< 11 (< 4.4%)	102 (0.3%)	0.5	12 (1.8%)	385 (0.4%)	**< 0.001**
Cardiac complications, *n* (%)	70 (28.2%)	2255 (5.7%)	**<0.001**	168 (24.9%)	7306 (7.8%)	**< 0.001**
Pulmonary complications, *n* (%)	17 (6.9%)	2863 (7.3%)	0.8	69 (10.2%)	8512 (9.0%)	0.3
Vascular complications, *n* (%)	< 11 (< 4.4%)	370 (0.9%)	**0.03**	< 11 (< 1.6%)	1806 (1.9%)	0.4
Gastrointestinal complications, *n* (%)	20 (8.1%)	3025 (7.7%)	0.8	75 (11.1%)	9705 (10.3%)	0.5
Infections, n (%)	< 11 (< 4.4%)	239 (0.6%)	0.2	11 (1.6%)	923 (1.0%)	0.1
Blood transfusions, *n* (%)	40 (16.1%)	2676 (6.8%)	**< 0.001**	116 (17.2%)	10,817 (11.5%)	**< 0.001**
Critical care therapy, *n* (%)	< 11 (< 4.4%)	628 (1.6%)	0.1	37 (5.5%)	3366 (3.6%)	**0.008**
In-hospital mortality, *n* (%)	< 11 (< 4.4%)	97 (0.2%)	0.1	< 11 (< 1.6%)	803 (0.9%)	0.6

Bold values indicate statistically significant *p* < 0.05

^a^Wilcoxon rank sum test; Pearson’s chi-squared test; Fisher’s exact test

### The Association Between History of Heart-Valve Replacement and Adverse Outcomes in Partial Nephrectomy Patients

In partial nephrectomy patients (*n* = 39,673), history of heart-valve replacement was associated with four of twelve adverse hospital outcomes (Table 3). Specifically, history of heart-valve replacement independently predicted cardiac complications [multivariable odds ratio (OR) 4.33, 95% CI 3.17–5.91; *p* < 0.001], blood transfusions (OR 2.00, 95% CI 1.42–2.82; *p* < 0.001), intraoperative complications (OR 1.53, 95% CI 1.04–2.26; *p* = 0.03), and longer length of stay [multivariable rate ratio (RR) 1.25, 95% CI 1.13–1.38; *p* < 0.001]. Conversely, no statistically significant association was recorded between history of heart-valve replacement and estimated hospital cost (*p* = 0.09), postoperative bleeding (*p* = 0.8), pulmonary complications (*p* = 0.3), vascular complications (*p* = 0.2), gastrointestinal complications (*p* = 0.7), infections (*p* = 0.5), critical care therapy use (*p* = 0.8), or in-hospital mortality (*p* = 0.5).

**Table 3:** Univariable and multivariable regression models addressing length of stay, perioperative complications, and in-hospital mortality according to history of heart-valve replacement in 134,563 kidney cancer patients undergoing partial or radical nephrectomy after adjustment for clustering at the hospital level using generalized estimating equation methodology

Outcomes of interest	Partial nephrectomy, *n* = 39,673				Radical nephrectomy, *n* = 94,890			
	Univariable		Multivariable*		Univariable		Multivariable*	
	RR/OR (95% CI)	*p* value	RR/OR (95% CI)	*p* value	RR/OR (95% CI)	*p* value	RR/OR (95% CI)	*p* value
Length of stay	**1.37** (1.24, 1.52)	**< 0.001**	**1.25** (1.13, 1.38)	**< 0.001**	**1.23** (1.16, 1.31)	**< 0.001**	**1.12** (1.05, 1.19)	**< 0.001**
Estimated hospital cost	**1.16** (1.05, 1.27)	**0.003**	1.09 (0.99, 1.20)	0.09	**1.11** (1.04, 1.19)	**0.001**	1.04 (0.98, 1.11)	0.2
Intraoperative complications	**1.84** (1.25, 2.71)	**0.002**	**1.53** (1.04, 2.26)	**0.03**	**1.78** (1.45, 2.19)	**< 0.001**	**1.53** (1.24, 1.89)	**< 0.001**
*Postoperative complications*								
Bleeding	1.56 (0.22, 11.24)	0.7	1.25 (0.17, 9.17)	0.8	**4.37** (2.46, 7.77)	**< 0.001**	**4.13** (2.31, 7.38)	**< 0.001**
Cardiac complications	**6.38** (4.86, 8.39)	**< 0.001**	**4.33** (3.17, 5.91)	**< 0.001**	**3.91** (3.29, 4.65)	**< 0.001**	**2.72** (2.25, 3.29)	**< 0.001**
Pulmonary complications	0.96 (0.60, 1.54)	0.9	0.77 (0.48, 1.23)	0.3	1.14 (0.89, 1.45)	0.3	0.92 (0.72, 1.18)	0.5
Vascular complications	**2.56** (1.12, 5.86)	**0.03**	1.85 (0.79, 4.32)	0.2	0.76 (0.41, 1.42)	0.4	0.62 (0.33, 1.17)	0.1
Gastrointestinal complications	1.04 (0.67, 1.62)	0.8	0.91 (0.58, 1.41)	0.7	1.08 (0.85, 1.37)	0.5	0.94 (0.74, 1.19)	0.6
Infections	1.99 (0.63, 6.26)	0.2	1.44 (0.46, 4.53)	0.5	1.64 (0.90, 2.99)	0.1	1.25 (0.68, 2.29)	0.5
Blood transfusions	**2.63** (1.88, 3.69)	**< 0.001**	**2.00** (1.42, 2.82)	**< 0.001**	**1.59** (1.30, 1.94)	**< 0.001**	**1.27** (1.03, 1.56)	**0.02**
Critical care therapy	1.80 (0.85, 3.81)	0.1	1.12 (0.52, 2.44)	0.8	**1.56** (1.12, 2.17)	**0.009**	1.14 (0.81, 1.60)	0.5
In-hospital mortality	3.29 (0.81, 13.38)	0.1	1.58 (0.39, 6.47)	0.5	1.22 (0.58, 2.57)	0.6	0.77 (0.36, 1.63)	0.5

Bold values indicate statistically significant *p* < 0.05

*Adjusted for age at admission, and comorbidities (Charlson comorbidity index)

*CI* Confidence interval, *RR* rate ratio, *OR* odds ratio

### Subgroup Analyses in Partial Nephrectomy Patients with History of Prosthetic Heart-Valve Replacement

In the subgroup of 39,619 partial nephrectomy patients, history of prosthetic heart-valve replacement independently predicted four of twelve adverse outcomes after partial nephrectomy, namely cardiac complications (OR 3.99, 95% CI 2.77–5.75; *p* < 0.001), blood transfusions (OR 2.16, 95% CI 1.47–3.16; *p* < 0.001), intraoperative complications (OR 1.67, 95% CI 1.09–2.56; *p* = 0.02), and longer length of stay (RR 1.29, 95% CI 1.15–1.44; *p* < 0.001; Table 4).

**Table 4:** Univariable and multivariable regression models addressing length of stay, perioperative complications, and in-hospital mortality according to history of prosthetic heart-valve replacement in 134,383 kidney cancer patients undergoing partial or radical nephrectomy after adjustment for clustering at the hospital level using generalized estimating equation methodology

Outcomes of interest	Partial nephrectomy, *n* = 39,619				Radical nephrectomy, *n* = 94,764			
	Univariable		Multivariable*		Univariable		Multivariable*	
	RR/OR (95% CI)	*p* value	RR/OR (95% CI)	*p* value	RR/OR (95% CI)	*p* value	RR/OR (95% CI)	*p* value
Length of stay	**1.41** (1.26, 1.58)	**< 0.001**	**1.29** (1.15, 1.44)	**< 0.001**	**1.26** (1.18, 1.34)	**< 0.001**	**1.15** (1.08, 1.23)	**< 0.001**
Estimated hospital cost	**1.18** (1.05, 1.32)	**0.005**	1.11 (0.99, 1.25)	0.07	**1.14** (1.06, 1.21)	**< 0.001**	**1.07** (1.01, 1.14)	**0.04**
Intraoperative complications	**1.97** (1.29, 3.02)	**0.002**	**1.67** (1.09, 2.56)	**0.02**	**1.79** (1.42, 2.25)	**< 0.001**	**1.56** (1.24, 1.97)	**< 0.001**
*Postoperative complications*								
Bleeding	2.00 (0.28, 14.40)	0.5	1.64 (0.22, 12.08)	0.6	**4.94** (2.71, 9.00)	**< 0.001**	**4.70** (2.56, 8.60)	**< 0.001**
Cardiac complications	**5.80** (4.23, 7.97)	**< 0.001**	**3.99** (2.77, 5.75)	**< 0.001**	**3.59** (2.95, 4.37)	**< 0.001**	**2.58** (2.07, 3.20)	**< 0.001**
Pulmonary complications	1.02 (0.61, 1.72)	0.9	0.83 (0.49, 1.40)	0.5	1.16 (0.89, 1.52)	0.3	0.96 (0.73, 1.26)	0.8
Vascular complications	**2.72** (1.10, 6.73)	**0.03**	1.99 (0.79, 5.05)	0.1	0.75 (0.38, 1.51)	0.4	0.63 (0.31, 1.26)	0.2
Gastrointestinal complications	1.07 (0.65, 1.76)	0.8	0.94 (0.57, 1.55)	0.8	1.08 (0.83, 1.40)	0.6	0.95 (0.73, 1.24)	0.7
Infections	2.55 (0.81, 8.04)	0.1	1.90 (0.60, 5.98)	0.3	1.47 (0.73, 2.96)	0.3	1.15 (0.57, 2.32)	0.7
Blood transfusions	**2.78** (1.91, 4.06)	**< 0.001**	**2.16** (1.47, 3.16)	**< 0.001**	**1.56** (1.25, 1.95)	**< 0.001**	**1.27** (1.01, 1.60)	**0.04**
Critical care therapy	1.97 (0.87, 4.46)	0.1	1.27 (0.55, 2.92)	0.6	**1.72** (1.21, 2.45)	**0.003**	1.29 (0.90, 1.86)	0.2
In-hospital mortality	**4.20** (1.03, 17.2)	**0.046**	2.09 (0.51, 8.59)	0.3	1.50 (0.71, 3.17)	0.3	0.99 (0.47, 2.10)	1.0

Bold values indicate statistically significant *p* < 0.05

*Adjusted for age at admission and comorbidities (Charlson comorbidity index)

*CI* Confidence interval, *RR* rate ratio, *OR* odds ratio

### Length of Stay, Estimated Hospital Cost, Perioperative Complications, and Mortality Rates in Radical Nephrectomy Patients

In radical nephrectomy patients (*n* = 94,890) with history of heart-valve replacement versus others, median length of stay was 5 versus 4 days (IQR 3–8 versus 3–6 days; *p* < 0.001; Table 2) and estimated median hospital cost were 47,439 versus 38,592 $USD (31,545–72,111 versus 26,036–60,917 $USD; *p* < 0.001). Addressing perioperative complications, 168 versus 7,306 (24.9 versus 7.8%; p < 0.001) experienced cardiac complications, 104 versus 8763 (15.3 versus 9.3%; *p* < 0.001) intraoperative complications, 75 versus 9705 (11.1 versus 10.3%; *p* = 0.5) gastrointestinal complications, 69 versus 8512 (10.2% versus 9.0%; *p* = 0.3) pulmonary complications, 12 versus 385 (1.8% versus 0.4%; p < 0.001) postoperative bleeding, 11 versus 923 (1.6% versus 1.0%; *p* = 0.1) infections, < 11 versus 1806 (< 1.6 versus 1.9%; *p* = 0.4) vascular complications, 116 versus 10,817 (17.2% versus 11.5%; *p* < 0.001) received blood transfusions, and 37 versus 3366 (5.5% versus 3.6%; *p* = 0.008) received critical care therapy. In-hospital mortality was recorded in < 11 versus 803 (<1.6 versus 0.9%; *p* =0.6) patients. For two of the above outcomes, actual counts and actual proportions are not shown due to NIS reporting rules. Instead, counts of less than eleven and the associated proportions are shown.

### The Association Between History of Heart-Valve Replacement and Adverse Outcomes in Radical Nephrectomy Patients

In radical nephrectomy patients (*n* = 94,890), history of heart-valve replacement was invariably associated with five of twelve adverse hospital outcomes (Table 3). Specifically, history of heart-valve replacement independently predicted postoperative bleeding (multivariable OR 4.13, 95% CI 2.31–7.38; *p* < 0.001), cardiac complications (OR 2.72, 95% CI 2.25–3.29; *p* < 0.001), intraoperative complications (OR 1.53, 95% CI 1.24–1.89; *p* < 0.001), blood transfusions (OR 1.27, 95% CI 1.03–1.56; *p* = 0.02), and longer length of stay (RR 1.12, 95% CI 1.05–1.19; *p* < 0.001). Conversely, no statistically significant association was recorded between history of heart-valve replacement and estimated hospital cost (*p* = 0.2), pulmonary complications (*p* = 0.5), vascular complications (*p* = 0.1), gastrointestinal complications (*p* = 0.6), infections (*p* = 0.5), critical care therapy use (*p* = 0.5), or in-hospital mortality (*p* = 0.5).

### Subgroup Analyses in Radical Nephrectomy Patients with History of Prosthetic Heart-Valve Replacement

In the subgroup of 94,764 radical nephrectomy patients, history of prosthetic heart-valve replacement independently predicted six of twelve adverse outcomes after radical nephrectomy, namely postoperative bleeding (OR 4.70, 95% CI 2.56–8.60; *p* < 0.001), cardiac complications (OR 2.58, 95% CI 2.07–3.20; *p* < 0.001), intraoperative complications (OR 1.56, 95% CI 1.24–1.97; *p* < 0.001), blood transfusions (OR 1.27, 95% CI 1.01–1.60; *p* = 0.04), longer length of stay (RR 1.15, 95% CI 1.08–1.23; *p* < 0.001), and higher estimated hospital cost (RR 1.07, 95% CI 1.01–1.14; *p* = 0.04; Table 4).

## Discussion

In kidney cancer patients undergoing partial or radical nephrectomy, the association between history of heart-valve replacement and length of stay, estimated hospital cost, perioperative complications, as well as in-hospital mortality is unknown. To address this knowledge gap, we relied on a population-based cohort of partial and radical nephrectomy patients within NIS (2000–2019) and made several important observations.

First, we identified important differences in descriptive characteristics between patients with versus without history of heart-valve replacement who underwent partial or radical nephrectomy. Specifically, partial and radical nephrectomy patients with history of heart-valve replacement were older (median partial nephrectomy 69 versus 60 years; radical nephrectomy 71 versus 63 years) and harbored higher comorbidity burden (CCI ≥ 3 partial nephrectomy 22 versus 12%; radical nephrectomy 32 versus 23%) than their respective counterparts. Considering these differences, it is essential to rely on multivariable adjustment for baseline patient characteristics in analyses focusing on adverse perioperative outcomes, as was done in the present study.

Second, we hypothesized that complication and in-hospital mortality rates following partial or radical nephrectomy in patients with versus without history of heart-valve replacement do not differ. Of those, in-hospital mortality is feared the most. Examination of in-hospital mortality revealed no statistically significant differences after either partial nephrectomy (< 4.4% versus 0.2%; *p* = 0.1) or radical nephrectomy (< 1.6% versus 0.9%; *p* = 0.6) and failed to achieve independent predictor status of higher in-hospital mortality in multivariable analyses (partial and radical nephrectomy *p* = 0.5). This finding is important in treatment decision-making regarding partial or radical nephrectomy in kidney cancer patients with history of heart-valve replacement. Particularly, healthcare providers can reassure patients with a history of heart-valve replacement that the risk of in-hospital mortality should not dissuade them from considering partial or radical nephrectomy as curative treatment option for kidney cancer.

Third, we also assessed eleven other adverse in-hospital outcomes in history of heart-valve replacement versus other partial or radical nephrectomy patients. In separately fitted multivariable models, history of heart-valve replacement independently predicted four of eleven adverse in-hospital outcomes in partial nephrectomy patients, namely intraoperative (OR 1.5; *p* < 0.001) and cardiac complications (OR 4.3; *p* < 0.001), blood transfusions (OR 2.0; *p* < 0.001), and longer length of stay (RR 1.3; *p* < 0.001). In radical nephrectomy patients, history of heart-valve replacement independently predicted five of eleven adverse in-hospital outcomes, namely postoperative bleeding (OR 4.1; *p* < 0.001), intraoperative (OR 1.5; *p* < 0.001) and cardiac complications (OR 2.7; *p* < 0.001), blood transfusions (OR 1.3; *p* = 0.02), and longer length of stay (RR 1.1; *p* < 0.001). Partial (*n* = 39,619) and radical nephrectomy (*n* = 94,764) subgroups, addressing the effect of prosthetic heart-valve replacement, virtually perfectly mimicked the results recorded in the overall cohort. Taken together, these observations indicate a less favorable in-hospital stay profile in patients with history of heart-valve replacement. However, this unfavorable profile is not prohibitive based on the absence of the ultimately feared in-hospital complication, namely in-hospital mortality.

Taken together, partial nephrectomy and radical nephrectomy patients with history of heart-valve replacement exhibit less favorable patient characteristics. These consist of older age and higher comorbidity burden (CCI ≥ 3). Despite these baseline disadvantages, heart-valve replacement patients undergoing partial or radical nephrectomy are not at higher risk of the most feared in-hospital complication, namely in-hospital mortality. Nonetheless, cardiac complications, blood transfusions, and intraoperative complications are significantly higher in those individuals. These observations are essential in medical decision-making and counselling prior to definitive therapy assignment. They should not discourage clinicians from relying on partial or radical nephrectomy as respective standards of care according to clinical indications for those two procedures.

The current study has limitations. First, due to the retrospective nature of NIS, selection and reporting biases may have remained. However, this limitation is shared with all previous analyses relying on NIS^4–7^ or other large-scale retrospective databases, such as Surveillance Epidemiology and End Results database.^7,16–18^ Second, despite its very large size, NIS only provides a limited number of patients with history of heart-valve replacement, due to the rarity of this condition. Therefore, subgroup analyses could only be performed in prosthetic heart-valve replacement but not in xenogenic or other heart-valve replacement patients. Moreover, we were unable to perform further subgroup analyses according to surgical approach (robotic-assisted versus laparoscopic versus open surgery). Third, NIS as well as ICD codes only offer a limited amount of detail. For example, timing, duration, and dose of anticoagulation as well as timing and frequency of blood transfusions were not available. Additionally, detailed information regarding procedure-related characteristics, such as intraoperative blood loss, were unknown. Moreover, we were unable to adjust for tumor characteristics since NIS does not contain such information. Finally, NIS exclusively provides in-hospital data. In consequence, data regarding readmissions and complications after hospital discharge were not available. Indeed, it could be interesting to also assess readmission and long-term complications rates after partial and radical nephrectomy in future studies.

## Conclusions

History of heart-valve replacement independently predicted four of twelve adverse outcomes in partial nephrectomy and five of twelve adverse outcomes in radical nephrectomy patients including intraoperative and cardiac complications, blood transfusions, and longer hospital stay. Conversely, no statistically significant differences were observed in in-hospital mortality.

### Supplementary Information

Below is the link to the electronic supplementary material.

Supplementary file1 (DOCX 20 kb)
