# Supplementary material for: Perioperative Complications and In-Hospital Mortality in Partial and Radical Nephrectomy Patients with Heart-Valve Replacement
Source: Ann Surg Oncol. 2024 Mar 25;31(8):5449–56. doi: 10.1245/s10434-024-15228-6 (PMC11236841; doi:10.1245/s10434-024-15228-6)
Supplement: Supplementary file 1 — Supplementary file1 (DOCX 20 kb) [file 10434_2024_15228_MOESM1_ESM.docx]

| **Supplementary Table 1:** International Classification of Disease (ICD) 9th revision Clinical Modification (ICD-9-CM), ICD 10th revision Clinical Modification (ICD-10-CM), as well as ICD 10th revision Procedure Coding System (ICD-10-PCS) codes used for identification of perioperative complications.   \| **Perioperative complication** \| **ICD-9 and ICD-10 codes** \| \| --- \| --- \| \| Intraoperative complications \| 997.02, 998.11, 998.12, 997.1, 998.2, 997.5, 997.49, 349.31, 997.99, 997.01, 997.09, 998.89, 997.39, 995.86, V58.89, 909.3, 999.9, 668.90, 995.22, 995.4, 995.89, 995.24, 995.0, 909.5, 998.11, 998.12,  G97.3xx, N99.61,N99.62, N99.71,N99.72, N99.81,K91.61, K91.62, K91.71, K91.72, K91.81, L76.01, L76.02, I97.410, I97.411, I97.418, I97.42, I97.51, I97.52, I97.711, I97.791, I97.810, I97.811, I97.88, G97.41, G97.48, G97.49, G97.81, M96.810, M96.811, M96.820, M96.821, M96.89,E36.01, E36.02, E36.11, E36.12, E36.8, J95.61, J95.62, J95.71, J95.72, J95.88, T883.xx, T884.xx, T88.5.xx, T88.6.xx, T88.7.xx, T88.8.xx, T88.9.xx, D78.0.xx, D78.1.xx, D78.81, M96.811. \| \| Postoperative bleeding \| 459.0, 958.2, 568.81,  R58, N99.820, N99.821, K66.1, E89.811, E89.821, D78.22, D78.32, D78.02, M96.841, M96.831. \| \| Cardiac complications \| 410.0, 410.1, 410.2, 410.3, 410.4, 410.5, 410.6, 410.7, 410.8, 410.9, 411.0, 402.11, 402.91, 428.0, 428.1, 428.21, 428.31, 428.41, 428.9, 427.5, 997.1,  I160, I161, I169, I20.xx, I21.xx, I22.xx, I23.xx, I24.xx, I44.xx, I48.xx, I46.2, I46.8, I46.9, I47.xx, I50.1, I50.21, I50.23, I50.31, I50.33, I50.41, I50.43, I50.811, I50.813, I50.82, I50.83, I50.84, I50.89, I50.9, I51.xx, I97.1.xx, I97.61.xx, I97.82, I97.89. \| \| Pulmonary complications \| 518.0, 518.4, 514, 466.0, 466.11, 466.19, 480.0, 480.1, 480.2, 480.3, 480.8, 480.9, 481, 482.0, 482.1, 482.2, 482.3, 482.4, 482.8, 482.9, 507.0, 518.81, 483.0, 483.1, 483.8, 485, 486, 418.81, 518.82, 799.1, 997.3,  J09.xx, J10.xx, J11.xx, J12.xx, J13.xx, J14.xx, J15.xx, J16.xx, J17.xx, J18.xx, J20.xx, J21.xx, J80.xx, J81.xx, J90, J91.xx, J93.xx, J96.00, J96.01, J96.02, J96.20, J96.21, J96.22, J96.9.xx, R09.2, J95.81.xx, J95.82.xx, J95.85.xx, J95.86.xx, J95.89. \| \| Vascular complications \| 415.1, 415.11, 415.12, 415.19, 451.2, 451.81, 451.9, 453.8, 453.9, 997.2, 999.2, 444.22, 444.81, 433, 433.0, 433.1, 433.2, 433.3, 433.8, 433.9, 434,434.0, 434.1, 434.9, 436, 437, 437.1, 437.2, 437.4, 437.3, 437.5, 437.6, 437.7, 437.8, 437.9,  I81, I82.210, I82.220, I82.23, I82.290, I82.4.xx, I82.6.xx, I82.A1.xx, I82.B.1.xx, I82.C1.xx, I82.8.xx, I82.890, I82.90, I80.xx, I71.xx, I77.7xx, I74.xx,I75.xx, I76, I26.xx, I28.8, I28.9. \| \| Gastrointestinal complications \| 531.0, 531.1, 531.2, 531.3, 532.0, 532.1, 532.2, 532.3, 540.0, 540.1, 540.9, 560.0, 560.1, 560.2, 560.3, 560.8, 560.9, 787.6, 997.4, 569.2, 569.3, 569.5, 569.6, 579.3, 008.45,  K25.0, K25.1, K25.3, K25.9, K26.0, K26.1, K26,3, K26.9, K27.0, K27.1, K27.3, K27.9, K28.0, K28.1, K28.3, K28.9, K35.xx, K36.xx, K45.xx, K65.xx, K66.xx, K67.xx, K68.xx, K61.0, K61.1, K62.6, K63.0, K63.1, K63.2, K63.3, K61.81.xx, K91.82, K91.83, K91.89. \| \| Infections \| 536.41, 519.01, 998.5, 993, 038, 054.5, 790.7, 995.91, 995.92,  T8140.xx, T8144.xx, A40.xx, A41.xx, B00.7, R78.81, R65.xx, A49,3, B96,0, B96.1, B96.6, B96.7, B96.89, B96.81. \| \| Blood transfusions \| 99.02, 99.03, 99.04, 99.05, 99.06, 99.07,  30233H0,30233H1,30233K0,30233K1, 30233L0, 30233L1,30233N0, 30233N1, 30233P0, 30233P1,30233R0, 30233R1, 30233T0, 30233T1. \| \| Critical care therapy \|  \| \| Invasive mechanical ventilation \| 967.0, 967.1, 967.25,  A1935Z, 5A1945Z, 5A1955Z. \| \| Percutaneous endoscopic gastrostomy tube insertion \| 431.1,  0DH63UZ, 0DH64UZ. \| \| Dialysis for acute kidney failure \| 584.5, 584.6, 584.7, 584.8, 584.9, N17.0, N17.1, N17.2, N17.8, N17.9 and  399.5, 5A1D70Z, 5A1D80Z, 5A1D90Z. \| \| Total parenteral nutrition \| 991.5,  3E0336Z, 3E0436Z, 3E0536Z, 3E0636Z. \| \| Tracheostomy \| 311, 312.1, 312.9,  0B110F4, 0B110Z4, 0B113F4, 0B113Z4, 0B114F4, 0B114Z4. \| |
| --- | --- | --- | --- | --- | --- | --- | --- | --- | --- | --- | --- | --- | --- | --- | --- | --- | --- | --- | --- | --- | --- | --- | --- | --- | --- | --- | --- | --- | --- | --- |
